# RETRACTED: Iandolo et al. Traditional and Recent Root Canal Irrigation Methods and Their Effectiveness: A Review. *Clin. Pract.* 2023, *13*, 1059–1072

**DOI:** 10.3390/clinpract14010004

**Published:** 2023-12-25

**Authors:** Alfredo Iandolo, Massimo Pisano, Alessio Buonavoglia, Francesco Giordano, Alessandra Amato, Dina Abdellatif

**Affiliations:** 1 Department of Medicine and Surgery, School of Dentistry, University of Salerno, 84081 Salerno, Italy; pisano.studio@virgilio.it (M.P.); giordano.francesco@vipnet.it (F.G.); aamato@unisa.it (A.A.); 2 Department of Biomedical and Neuromotor Sciences, School of Dentistry, University of Bologna, 40125 Bologna, Italy; alessio.buonavoglia2@unibo.it; 3 Department of Endodontics, Faculty of Dentistry, University of Alexandria, Alexandria 21531, Egypt; dinaabdellatif81@gmail.com

The *Clinics and Practice* Editorial Office retracts the article titled “Traditional and Recent Root Canal Irrigation Methods and Their Effectiveness: A Review” [1].

Following publication, concerns were brought to the attention of the Editorial Office regarding a significant overlap with a previously published review [2] from a different authorship group and without appropriate citation.

Adhering to our complaints procedure, an investigation was conducted by the Editorial Office and Editorial Board which confirmed the extensive use and dispersed nature of the overlap. Therefore, the *Clinics and Practice* Editorial Office has taken the decision to retract [1].

This retraction was approved by the Editor in Chief of the journal *Clinics and Practice.*

The authors did not provide a comment on this retraction decision.
